# SVHRSP Alleviates Age-Related Cognitive Deficiency by Reducing Oxidative Stress and Neuroinflammation

**DOI:** 10.3390/antiox13060628

**Published:** 2024-05-21

**Authors:** Yingzi Wang, Zhenhua Wang, Songyu Guo, Qifa Li, Yue Kong, Aoran Sui, Jianmei Ma, Li Lu, Jie Zhao, Shao Li

**Affiliations:** 1 Department of Physiology, College of Basic Medical Sciences, Liaoning Provincial Key Laboratory of Cerebral Diseases, Dalian Medical University, Dalian 116044, China; wangyz0119@163.com (Y.W.); 18202653929@163.com (Z.W.); gsy002@outlook.com (S.G.); liqifa@dmu.edu.cn (Q.L.); kongyue971111@163.com (Y.K.); segasar123@outlook.com (A.S.); 2 Department of International Medical Services, Second Affiliated Hospital of Dalian Medical University, Dalian 116023, China; 3 National-Local Joint Engineering Research Center for Drug-Research and Development (R&D) of Neurodegenerative Diseases, Dalian Medical University, Dalian 116044, China; 4 Department of Anatomy, College of Basic Medical Sciences, Dalian Medical University, Dalian 116044, China; ma_jianmei@hotmail.com; 5 Department of Anatomy, College of Basic Medical Sciences, Shanxi Medical University, Taiyuan 030001, China

**Keywords:** SVHRSP, cognitive deficiency, oxidative stress, neuroinflammation, Sirt 1 pathway

## Abstract

Background: Our previous studies have shown that scorpion venom heat-resistant synthesized peptide (SVHRSP) induces a significant extension in lifespan and improvements in age-related physiological functions in worms. However, the mechanism underlying the potential anti-aging effects of SVHRSP in mammals remains elusive. Methods: Following SVHRSP treatment in senescence-accelerated mouse resistant 1 (SAMR1) or senescence-accelerated mouse prone 8 (SAMP8) mice, behavioral tests were conducted and brain tissues were collected for morphological analysis, electrophysiology experiments, flow cytometry, and protein or gene expression. The human neuroblastoma cell line (SH-SY5Y) was subjected to H_2_O_2_ treatment in cell experiments, aiming to establish a cytotoxic model that mimics cellular senescence. This model was utilized to investigate the regulatory mechanisms underlying oxidative stress and neuroinflammation associated with age-related cognitive impairment mediated by SVHRSP. Results: SVHRSP significantly ameliorated age-related cognitive decline, enhanced long-term potentiation, restored synaptic loss, and upregulated the expression of synaptic proteins, therefore indicating an improvement in synaptic plasticity. Moreover, SVHRSP demonstrated a decline in senescent markers, including SA-β-gal enzyme activity, P16, P21, SIRT1, and cell cycle arrest. The underlying mechanisms involve an upregulation of antioxidant enzyme activity and a reduction in oxidative stress-induced damage. Furthermore, SVHRSP regulated the nucleoplasmic distribution of NRF2 through the SIRT1-P53 pathway. Further investigation indicated a reduction in the expression of proinflammatory factors in the brain after SVHRSP treatment. SVHRSP attenuated neuroinflammation by regulating the NF-κB nucleoplasmic distribution and inhibiting microglial and astrocytic activation through the SIRT1-NF-κB pathway. Additionally, SVHRSP significantly augmented Nissl body count while suppressing neuronal loss. Conclusion: SVHRSP could remarkably improve cognitive deficiency by inhibiting oxidative stress and neuroinflammation, thus representing an effective strategy to improve brain health.

## 1. Introduction

The global elderly population in human societies is currently undergoing a substantial growth, giving rise to the emergence of aging as a prominent and pressing issue. Advancing age is a significant risk factor in the development of neurodegenerative disorders. The brain is a primary site impacted by the aging process, leading to gradual cognitive decline, which imposes significant stress and economic burden on families [1,2,3]. The development of efficient therapeutic tools to retard the aging processes and increase healthy life expectancy has become a prominent concern in the medical field, leading to an increasing involvement of scholars.

Senescence-accelerated mouse prone 8 (SAMP8) is an accelerated aging model that was established through phenotypic selection from a genetically homogeneous pool of the AKR/J strain of mice [4,5]. The SAMP8 model strain exhibits kyphosis, sparse hair, depressive-like behavior, and impaired mobility from the age of 5 months onwards, resembling characteristics commonly observed in elderly individuals, particularly those experiencing cognitive decline. The efficacy of the SAMP8 model in simulating the pathogenesis of age-related memory disorders has been substantiated by multiple studies. Currently, this animal model is globally recognized as an exceptional tool for investigating aging.

The enzyme SIRT1, also known as sirtuin1 (silencing information regulator 2 related enzyme 1) is an NAD+-dependent deacetylase. It has been reported that upregulating the expression of the SIRT1 gene can extend the lifespan of various organisms, including yeast [6], flies [7], worms [8], and mammals [9]. The enzyme SIRT1 deacetylates its substrates, such as P53, FOXOs, PGC1-a, PPAR-γ, and NF-κB [10,11], thereby modifying their transcriptional activities and protein levels that are involved in the regulation of senescence [12,13].

The scorpion is utilized in traditional Chinese medicine for the treatment of conditions such as epilepsy, bradykinesia, and dementia. Our laboratory has synthesized a patented product known as scorpion venom heat-resistant synthesized peptide (SVHRSP) [14]. This peptide is extracted and purified from the active single component found in scorpion venom, with its amino acid sequence determined through mass spectrometry analysis. With its small molecular weight and ability to penetrate the blood–brain barrier, SVHRSP shows promise as a potential neuroprotective drug. Previous studies have demonstrated that SVHRSP can extend the lifespan of Caenorhabditis elegans (C. elegans) by activating the IGF-1-repressed anti-aging nuclear factor Daf16/FOXO [15]. Additionally, SVHRSP has exhibited a potentiation of motor function in a mouse model of 6-OHDA-induced Parkinson’s disease (PD) [16]. These findings suggest that SVHRSP may confer beneficial effects on cerebral aging. 

According to our current research findings, we hypothesize that SVHRSP has the potential to mitigate the process of aging. Here, we propose that SVHRSP may exert its anti-aging effects by modulating the SIRT1 signaling pathway and mitigating age-related cognitive decline.

## 2. Materials and Methods

### 2.1. Materials

Scorpion venom heat-resistant peptide (SVHRP, national invention patent ZL01106166.9) was extracted from the East Asian pincer scorpion using a specialized process. The single-component heat-stable synthetic peptide of scorpion venom, SVHRSP, was subsequently obtained through solid-phase chemical synthesis and further purified through chromatography. Its amino acid sequence was determined by using mass spectrometry and is documented in the patent on the chemical composition and application of SVHRSP (MW = 1524.7 d) (patent number: ZL2016 1 0645111.7).

### 2.2. Animals and Treatments

The four-month-old senescence-accelerated mouse resistant 1 (SAMR1) mice (*n* = 10) and senescence-accelerated mouse prone 8 (SAMP8) mice (*n* = 10) were obtained from the Peking University Health Science Center. The animals were housed in a controlled room at a temperature of 22 °C ± 2 °C, with a 12 h light/dark cycle and ad libitum access to food and water. During the course of the study, the mice were treated with either a vehicle or SVHRSP for a duration of 12 weeks. The treatment involved daily intraperitoneal injections of SVHRSP at a concentration of 50 μg/kg. At week 12, behavioral tests including Y maze, passive avoidance experiment, novel object recognition, and Morris water maze were conducted. At the end of week 16, the mice were euthanized and their brains were rapidly separated and either frozen using liquid nitrogen before being stored at −80 °C or fixed in 4% paraformaldehyde solution (PFA). All experimental procedures involving animals followed the guidelines outlined by the Chinese National Research Council Guide for Care and Use of Laboratory Animals and received approval from the Institutional Ethics Committee of Dalian Medical University (ethics committee approval permit no. AEE23046). Every effort was made to minimize both the number of experimental animals used as well as any potential suffering they may have experienced. 

### 2.3. The Y Maze 

The Y-maze task was conducted to evaluate short-term spatial learning and memory. The structure consisted of a three-arm maze enclosed by walls in the shape of a capital Y, with each arm maintaining an identical angle relative to the central trunk. During the test, mice were placed at one end of an arm and allowed to freely explore the maze for 8 min. The video tracking analysis system recorded the percentage of alternating behaviors, indicating when the mouse crossed between all three arms. The Y-maze test was performed following the methodology described by Van der Borght et al. [17].

### 2.4. Passive Avoidance Tests

The passive avoidance experiment is a behavioral method designed to assess the learning and memory abilities of mice by exploiting their natural inclination to avoid light and external stimuli, thereby utilizing a dark area that can administer electric shocks and a lit area separated by a central door in the passive avoidance chamber [18]. Upon complete entry into the dark section, mice receive an immediate 3 s electric shock of 20 mA before returning to the open box where they automatically stop. This event serves as the acquisition phase for memory formation. The incubation period refers to the time taken for mice to re-enter either the open or dark compartments. Subsequently, after removing the mice from the chamber, both boxes are disinfected with alcohol to eliminate any residual odor. After 24 h, the test is repeated wherein the central channel is opened and observations are made on entering into darkness and receiving shocks within a test duration of 5 min. The measurements obtained from this passive avoidance test were analyzed following Tsai et al.’s methodology [19].

### 2.5. Novel Object Recognition (NOR) 

The novel object recognition (NOR) task was employed to evaluate the non-spatial learning and memory abilities in mice associated with the hippocampus [11]. The experimental apparatus comprised an opaque plastic box measuring 50 cm in length, width, and height. Prior to the experiment, animals were familiarized with the environment for 30 min. In the sample phase of the first trial, two identical objects were placed inside the chamber, allowing the mice to explore for a duration of 5 min. Following a 24 h interval, during the second task, one object was replaced by another and exploration time was recorded again for 5 min. The recognition index (RI) was calculated by dividing the time spent exploring the novel object by the total exploration time. Conversely, the discrimination index (DI) was determined by dividing the difference in exploration times between novel and old objects by the total exploration time [20,21].

### 2.6. Morris Water Maze

The Morris water maze (MWM) is a method used to evaluate spatial learning and memory abilities in mice by immersing them in water and challenging them to locate a hidden platform [22]. The circular pool, measuring 120 cm in diameter, is filled with water maintained at a temperature of 22 ± 2 °C using an integrated heating system. It is virtually divided into four equal quadrants: northeast, northwest, southeast, and southwest. The transparent plastic platform with a diameter of 12 cm is positioned approximately 2–3 cm below the water surface within the third quadrant, making it difficult to detect from above. Mice are consistently placed at the same starting position for each trial. A trial ends when the mouse successfully reaches the platform (escape latency) or after 60 s if unsuccessful. In cases where mice fail to find the platform within this time frame, they are guided toward it manually. Following each trial, mice remain on the platform for 15 s before proceeding further testing procedures. To assess memory retention, probe trials are conducted on the final day of testing (6th day). During these trials, the platform is removed from the pool and mice are allowed to swim freely for 60 s while their percentage of time spent in the target quadrant is recorded as an indicator of memory performance. All trials are monitored using an overhead video camera connected to Etho-Vision XT 11.5 Experiment software system. 

### 2.7. Electrophysiology Experiments

The most commonly employed method for studying synaptic plasticity is long-term potentiation (LTP), which is widely recognized as the molecular foundation of learning and memory. Conversely, impaired LTP typically indicates dysfunction in learning and memory processes. In this experiment, electrophysiological techniques were utilized to investigate the impact of SVHRSP on synaptic plasticity in SAMP8 mice by examining the hippocampus. Stimulation was applied to mossy fibers in the DG region while recording field potentials in CA1. The specific procedure involved selecting 3 mice randomly from each group after Morris water maze testing, followed by severing their brains on ice into slices with a thickness of 300–400 μm. High-frequency electrical stimulation (HFS) at 100 Hz with a duration of 1000 ms × 2 was used to induce LTP in Schaffer collateral axons within the CA1 area, with each dose administered at intervals of 20 s. Successful induction of LTP was determined if there was an increase in excitatory postsynaptic potential (EPSP) exceeding 20% that lasted for more than 30 min. The slope of EPSP onset and the magnitude of LTP enhancement were measured and compared.

### 2.8. The Technique of Transmission Electron Microscopy

The synaptic ultrastructure of the hippocampal CA1 region was examined by using transmission electron microscopy. The left-brain tissue was fixed in 2.5% glutaraldehyde for 24 h and sectioned into 1 mm cubes. Subsequently, the samples were treated with 1% osmium tetroxide for 2–3 h, washed twice with phosphate-buffered saline (PBS), dehydrated in a series of graded ethyl alcohol and acetone solutions, and finally embedded in a mixture of pure acetone and embedding liquid (at a ratio of 2:1). Ultrathin 50 nm thick sections were cut and desiccated. The sections were subsequently subjected to double staining with 2% uranyl acetate and 2% lead citrate for a duration of 15 min each at ambient temperature. Following rinsing and drying, the synaptic ultrastructure within the hippocampal CA1 region was observed under a transmission electron microscope, capturing four images per section. The standard synaptic morphology was characterized by the presence of intact synaptic vesicles and synaptic cleft.

### 2.9. Nissl Staining

The Nissl staining technique was employed to visualize neuronal morphology, as previously described. Following the neurobehavioral assessment, brain tissues were collected and fixed in 4% polyformaldehyde solution, followed by routine procedures of dehydration, embedding, and microtome sectioning (10 μm). Subsequently, the sections were dewaxed and rehydrated before being stained with 1% cresyl violet (Beyotime Biotechnology, Shanghai, China) for a duration of 5 min. The specimens were washed three times with pre-cooled triple-distilled water for 2 min each. Dehydration was carried out in a stepwise manner using 70% (2 min), 90% (2 min), and 100% (5 min) alcohol. Subsequently, they were rinsed twice in xylene for one minute each before being sealed with neutral resin. Photomicrographs of the entorhinal cortex and hippocampus were captured.

### 2.10. Assessment of the Activity of Senescence-Associated β-Galactosidase (SA-β-gal)

The activity of SA-β-gal in hippocampus tissue sections and cell samples was determined using the Senescence β-Galactosidase Staining Kit (Beyotime Biotechnology Co., Ltd., Shanghai, China). The SA-β-gal-positive cells (displaying a blue color) were stained and quantified utilizing Image-Pro Plus 6.0 software.

### 2.11. Immunohistochemical and Immunofluorescence Staining

The brain tissue was fixed by means of immersion in a buffered solution of 4% paraformaldehyde and subsequently dehydrated, cleared, and embedded. Sections were then cut using an RM2125 microtome (Leica Biosystems, Nussloch, Germany). The sections were dewaxed with xylene and hydrated in an ethanol gradient. Pretreatment of the sections involved incubation with 3% H_2_O_2_ for 10 min at 37 °C. Following this, the sections were incubated overnight at 4 °C with rabbit anti-GFAP primary antibody (Abcam, Cambridge, UK, ab7260, diluted at 1/200) or goat anti-Iba1 primary antibody (Abcam, ab5076, diluted at 1/200). On the next day, the sections were washed three times with PBS and then incubated at room temperature for 30 min with HRP-labeled goat anti-rabbit IgG polymer (PV-9001; Zhongshan Golden Bridge Biotechnology, Beijing, China; diluted at 1/100) or HRP-labeled rabbit anti-goat IgG polymer (PV-9003; Zhongshan Golden Bridge Biotechnology; diluted at 1/100). Visualization was achieved using 0.05% DAB (ZLI-9018; Zhongshan Golden Bridge Biotechnology), followed by image capture using an Olympus microscope (Tokyo, Japan) and analysis through Image J 1.0x software (NIH, Bethesda, MD, USA). The cell was cultured in 24-well plates, with or without H_2_O_2_ treatment for 24 h. Subsequently, the medium was aspirated and washed three times with 0.01M PBS. The cells were then fixed with pre-cooled 4% PFA for 15 min. After that, they were washed three times with 0.3% Triton-100 for 15 min and blocked with 5% BSA at room temperature for one hour. Following this, the sections were incubated overnight at a temperature of 4 °C with anti-NRF2 primary antibody (Abcam, diluted at 1/200). On the next day, the secondary antibody was incubated at room temperature. Once sealed, images were captured using an Olympus microscope (Tokyo, Japan) and analyzed using Image J 1.0x software (NIH, Bethesda, MD, USA).

### 2.12. SOD, MDA, and GSH Assay

The brain tissues were extracted and rinsed three times in cold isotonic saline solution (0.9%). Subsequently, the tissues were homogenized using cold Tris-HCl buffer (pH 7.4) to obtain a 10% homogenate. Malondialdehyde (MDA) reacts with thiobarbituric acid (TBA) under acidic conditions, resulting in the formation of a pink-colored complex that can be measured spectrophotometrically at a wavelength of 532 nm [23]. The plasmatic MDA level was expressed as nmol MDA/mg of protein. The oxidation of glutathione (GSH) by the sulfhydryl reagent 5,5′-dithio-bis (2-nitrobenzoic acid) (DTNB) produces a yellow derivative known as 5′-thio-2-nitrobenzoic acid (TNB) [24]. This reaction was measured at 412 nm and expressed as nmol GSH/mg of protein by reference to a standard curve. Superoxide dismutase (SOD) activity was evaluated using the method developed by Beauchamp and Fridovich [25], which is based on its ability to inhibit the photochemical reduction of nitro blue tetrazolium by superoxide anion. The resulting blue-colored formazan product was monitored at 580 nm. One unit of SOD activity corresponds to a 50% inhibition rate in NBT photoreduction. 

### 2.13. Cell Culture and Treatments

The SH-SY5Y cell line was cultured in fresh DMEM supplemented with 10% serum and incubated at 37 °C with 5% CO_2_ for cell experiments. The cells were divided into four groups: control group, SVHRSP-alone group, H_2_O_2_ group, and H_2_O_2_ plus SVHRSP group. For experimental use, the cells were resuscitated and passaged to the third generation. In the control group, only cells were added without any additional treatment. The SVHRSP-alone group was treated with a concentration of 20 μM SVHRSP for a duration of 6 h. The H_2_O_2_ group received a treatment of 200 μM H_2_O_2_ for 24 h. In the H_2_O_2_ plus SVHRSP group, pretreatment with 20 μM SVHRSP was conducted for 6 h followed by exposure to 200 μM H_2_O_2_ for 24 h. Cells from each respective group were collected for subsequent experiments.

### 2.14. Cell Cycle Assay

After rinsing the treated SH-SY5Y neuronal cells with the PBS buffer, the cells were fixed overnight at 4 °C in 70% pre-chilled ethanol. Subsequently, the cells were stained with propidium iodide (PI) and RNase A for a duration of 30 min. Lastly, the cell cycle analysis of treated SH-SY5Y neuronal cells was performed by using BD flow cytometry and the data were analyzed utilizing Cell Quest Pro (version 5.1) software.

### 2.15. Enzyme-Linked Immunosorbent Assay (ELISA)

The levels of *IL-6*, *IL-1B*, and *TNF-α* were quantified using commercially available ELISA kits. This assay employs the quantitative sandwich immunoassay technique to accurately measure the concentration of these cytokines. The standard curve established a direct correlation between optical density (OD) values and the number of secreted cytokines.

### 2.16. RNA Extraction and Relative Quantification Using Real-Time Polymerase Chain Reaction (qRT-PCR)

The total RNA was extracted using an RNA Easy kit following the manufacturer’s instructions (Bioecon Biotec Co., Shanghai, China). The purity of RNA samples was assessed by measuring the OD260/280. cDNA synthesis was performed through reverse transcription using StarScript III All-in-one RT Mix with gDNA Remover (GenStar, Beijing, China). Real-time PCR (A301-10, 2xRealStar Fast SYBR Qpcr Mix, GenStar, Beijing, China) was employed to detect the gene expression levels of *TNF-α*, *IL-6*, and *IL-1B*. The PCR analysis was carried out using a Mastercycler ep realplex apparatus (Eppendorf, Germany). Primer sets for *TNF-α*, *IL-6*, *IL-1B*, and GAPDH were purchased from Dingguo Changsheng Biotechnology Co. (Beijing, China).

### 2.17. Western Blot

The tissue was homogenized using a commercially available lysis buffer (Cell Signaling) supplemented with a cocktail of protease and phosphatase inhibitors (Sigma-Aldrich, P8340, Shanghai, China). The homogenates were sonicated and centrifuged at 4 °C for 5 min at 16,000× *g*. Supernatants were collected, and the protein concentration was determined using the Pierce BCA Protein Assay Kit (Thermo Scientific, 23227, Waltham, MA, USA). Equal amounts (30 μg) of total protein from each cellular extract were loaded onto SDS-PAGE gels and subjected to Western blotting. Proteins were separated on 4–15% gradient precast polyacrylamide gels (Mini-PROTEAN TGX StainFree Protein Gels, BioRad, Hercules, CA, USA), followed by electrophoretic transfer to PVDF membranes (Millipore, Shanghai, China). The membranes were then incubated in blocking buffer (Invitrogen, Carlsbad, CA, USA) for 30 min at room temperature and subsequently overnight at 4 °C with primary antibodies including SYN (Abcam, ab32127, Cambridge, UK), PSD95 (ABclonal, A7889, Wuhan, China), NRF2 (Abcam, ab137550, Cambridge, UK), HO-1 (Abcam, ab189491, Cambridge, UK), SOD1 (Cell Signaling Technology, 2770, Danvers, MA, USA), P16 (Proteintech, Wuhan, China), P21 (Proteintech, 10355-1-AP, Wuhan, China), P53 (Abcam, ab26, Cambridge, UK), SIRT1 (WanleiBio, WL00599, Shenyang, China), NF-kB p65 (Cell Signaling Technology, Danvers, MA, USA, D14E12), JNK (Sigma-Aldrich, SAB4200176, Shanghai, China), P-JNK (Cell Signaling Technology, 9251, Danvers, MA, USA), P38 (Cell Signaling Technology,9212, Danvers, MA, USA), P-P38 (Cell Signaling Technology,4511, Danvers, MA, USA), LaminB1 (Cell Signaling Technology, 13435, Danvers, MA, USA), and GADPH (WanleiBio, WL01114, Shenyang, China). After washing the membranes, signals were detected by using commercial kits (Western Breeze, Invitrogen Carlsbad, CA, USA) containing either anti-rabbit or anti-mouse secondary antibodies conjugated to alkaline phosphatase along with the corresponding chemiluminescent substrate following the manufacturer’s instructions. Finally, membranes were developed using Chemidoc Touch Imaging System 732BR1030 (Bio-Rad, Hercules, CA, USA).

### 2.18. Statistics Analysis

Cell count, fluorescence intensity, and Western blot band gray values were analyzed by using Image J 1.0x, and the results were expressed as means ± SEM and analyzed with GraphPad Prism 9.0 (GraphPad Software, San Diego, CA, USA). The differences among the groups were statistically analyzed by Student’s *t*-test, to compare means between two groups, and one-way analysis of variance (one-way ANOVA). In all comparisons, *p* < 0.05 was considered statistically significant.

## 3. Results

### 3.1. SVHRSP Reduced Memory Impairment and Improved Synaptic Functions

**Figure 1:**
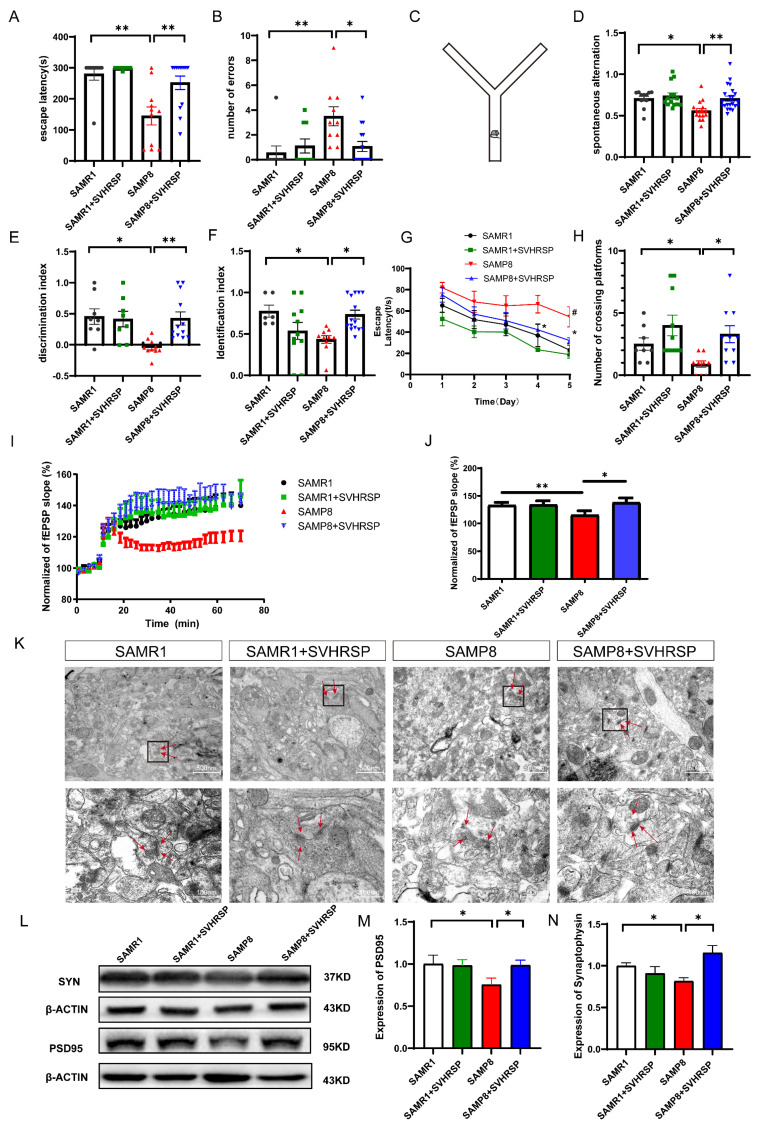
SVHRSP reduced memory impairment and improved synaptic functions. (**A**–**H**) The effects of SVHRSP on behavioral testing. (**A**) The escape latency of the passive avoidance experiment; (**B**) the number of mice that entered the dark room in the passive avoidance test; (**C**) a device structure-style diagram of the Y Maze; (**D**) the percentage of spontaneous alternate behavior in the Y-maze trial; (**E**) the discrimination index of each group of mice in the NOR test; (**F**) the identification index of each group of mice in the NOR test; (**G**) the escape latency to reach the platform in the MWM training phase; (**H**) the number of mice crossing the platform in the MWM probe trials; (**I**,**J**) The effects of SVHRSP on long-term potentiation including slope change and amplitude change; (**K**) The effects of SVHRSP on the synaptic ultrastructure in the hippocampus; The red arrows indicate the presence of synaptic vesicles and postsynaptic dense bodies. (**L**–**N**) the representative Western blot bands and the quantification of relative protein expression for SYN and PSD95. The bars represent the mean ± SD. * *p* < 0.05, ** *p* < 0.01, ^#^ *p* < 0.05, versus the indicated groups.

To investigate the effect of SVHRSP on cognitive impairments of SAMP8 mice, we treated SAMP8 mice with SVHRSP daily for 20 days, followed by conducting the passive avoidance test, Y-maze trial, the novel object recognition test (NOR), and the Morris water maze (MWM) trials (Figure 1A–H). Compared to the SAMR1 group, the SAMP8 group exhibited a shorter escape latency and a higher number of errors. The escape latency time of SAMP8 mice treated with SVHRSP was longer than that of untreated SAMP8 mice, indicating an improvement in fear memory impairment (Figure 1A,B). The results of the Y-maze test revealed that SAMP8 mice exhibited impaired recognition and memory abilities for novel environments compared to SAMR1 mice, with fewer spontaneous alternations into each arm. However, the SVHRSP-treated SAMP8 group showed increased spontaneous alterations into each arm (Figure 1D), suggesting an improvement in memory ability for aging mice. Additionally, in the NOR test, there was a significant increase observed in both the number of novel object recognitions and the exploration time for SAMP8 mice within the drug treatment group, thereby indicating an improved recognition ability (Figure 1E,F). Furthermore, water maze experiments demonstrated that administration of SVHRSP to SAMP8 mice significantly shortened their incubation period and enhanced their retention time in the target quadrant on the fourth and fifth days of training during platform navigation (Figure 1G,H). 

LTP was employed to assess the impact of SVHRSP on synaptic plasticity in SAMP8 mice. The fEPSP augmentation in the statistical field following high-frequency stimulation was attenuated in SAMP8 mice, whereas it was reversed after the administration of SVHRSP (Figure 1I,J). The findings indicate that SVHRSP has the potential to enhance synaptic plasticity in the hippocampus of SAMP8 mice. Under electron microscopy (Figure 1K), the synaptic ultrastructure of the SAMR1 group remained intact, displaying a clearly identifiable presynaptic membrane, synaptic cleft, and postsynaptic membrane. The presynaptic membrane exhibited a substantial quantity of spherical synaptic vesicles while simultaneously demonstrating postsynaptic density. In contrast, within the SAMP8 group, the synaptic cleft appeared blurred, with a reduced number of synaptic structures, and a significantly decreased quantity of synaptic vesicles along with thinner postsynaptic density. Compared to the SAMP8 group, the SAMP8 plus SVHRSP group exhibited an augmentation in synaptic vesicle count and a thickening of postsynaptic density, indicating successful restoration of the synaptic structure following SVHRSP intervention (Figure 1K). The Western blot analysis conducted on synapse-associated proteins revealed decreased expression levels for synapsin (SYN) and postsynaptic density 95 (PSD95) proteins within the hippocampi of SAMP8 mice when compared to those of SAMR1 mice; however, the intervention with SVHRSP resulted in a significant upregulation for SYN and PSD95 protein expression levels (Figure 1L–N). The findings suggest that SVHRSP has the potential to mitigate cognitive decline and enhance synaptic plasticity in SAMP8 mice.

### 3.2. SVHRSP Downregulated the Expression of Aging Markers

**Figure 2:**
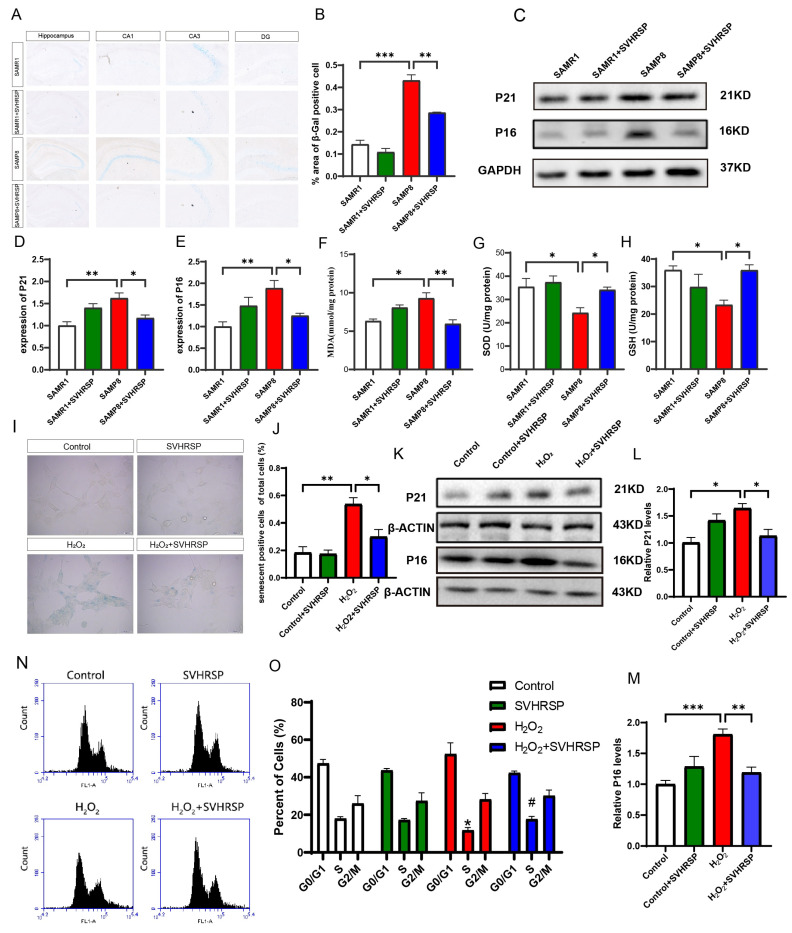
SVHRSP downregulated the expression of aging markers. (**A**,**B**) Representative SA-β-gal staining images and quantitative analysis in SAMP8 mice. (**C**) Representative WB bands. (**D**,**E**) Quantitative analysis of P21 and P16 in vivo. (**F**–**H**) Quantitative analysis of GSH-PX, SOD, and MDA enzyme activity in vivo. (**I**,**J**) Representative SA-β-gal staining images and quantitative analysis in H_2_O_2_-induced SH-SY5Y cells. (**K**) Representative WB bands. (**L**,**M**) Quantitative analysis of P21 and p1 in vitro. (**N**,**O**) Quantitative analysis of cell cycle arrest in the G0/G1 phase. The cell fraction in the G0/G1 phase, G2/M phase, and S phase was calculated. The bars represent the mean ± SD. * *p* < 0.05, ** *p* < 0.01, *** *p* < 0.001, ^#^ *p* < 0.05, versus the indicated groups (*n* = 3 for each group).

Cellular senescence is a crucial manifestation of the aging process. The primary characteristic of cellular senescence is an elevation in lysosomal β-galactosidase activity. To observe changes in the number of senescent cells in the brains of SAMP8 mice, we utilized β-Gal galactosidase staining to mark these cells. Our findings reveal that the quantity of senescence-positive cells was significantly higher in the brains of SAMP8 mice compared to SAMR1 mice. Additionally, a significant decrease in the number of senescence-positive cells was observed within the SAMP8 treatment group compared to the SAMP8 model group (Figure 2A,B). These findings strongly indicate that SVHRSP exhibits the potential to decrease cell senescence in the brain and impede processes associated with brain aging.

The phenomenon of cellular senescence is characterized by cell cycle arrest and cessation of cell division. The proteins P16 and P21 function as markers for cell cycle arrest and are considered important indicators for cellular senescence. The Western Blot analysis demonstrated higher expression levels of P16 and P21 proteins in the brains of SAMP8 mice compared to SAMR1 mice. Conversely, when compared with the SAMP8 model group, significant reductions were observed in the expression levels of P16 and P21 proteins within the SAMP8 treatment group, indicating that SVHRSP could effectively reduce expression levels associated with aging markers within mouse brains (Figure 2C–E). The relationship between aging and oxidative stress is inseparable. 

With advancing age, there is an upsurge in the body’s production of oxygen free radicals, coupled with a deceleration in metabolism, resulting in an imbalance between radical generation and elimination processes, ultimately culminating in oxidative stress. Glutathione peroxidase (GSH-Px) and superoxide dismutase (SOD) are two crucial antioxidant enzymes in the body, whereas malondialdehyde (MDA) is a product of lipid peroxidation caused by free radicals. MDA exhibits cytotoxicity. The findings indicate that compared to the SAMR1 control group, the brains of SAMP8 mice showed decreased levels of two antioxidant enzymes—glutathione peroxidase (GSH-PX) and superoxide dismutase (SOD). However, the treatment of SVHRSP in SAMP8 mice significantly enhanced the activities of these two antioxidant enzymes. Although there was an increase in MDA toxicity in the SAMP8 model group compared to the SAMR1 control group, this difference did not reach statistical significance. Nevertheless, treatment of SVHRSP in SAMP8 mice led to a reduction in MDA activity and mitigated oxidative damage to some extent (Figure 2F–H).

Subsequently, we employed hydrogen peroxide (H_2_O_2_) to induce cellular senescence in SH-SY5Y cells as a simulation model. The excessive generation of reactive oxygen species (ROS) in vivo and their failure to be timely eliminated result in oxidative stress, which subsequently leads to DNA damage that is irreparable and ultimately culminates in cell senescence. Our findings demonstrate that intracellular levels of reactive oxygen species (ROS) were significantly elevated in the H_2_O_2_ group compared to the control group (Figure S1). Pretreatment with SVHRSP effectively attenuated ROS production, as evidenced by a significant reduction in fluorescence intensity when compared to the H_2_O_2_ group (Figure S1). These results indicate that H_2_O_2_ can induce oxidative stress in SH-SY5Y cells. Furthermore, the proportion of senescent cells was higher in the H_2_O_2_ group than in the control group. However, after pretreatment with SVHRSP, there was a significant decrease in the number of positive senescent cells compared to the H_2_O_2_ group (Figure 2I,J). These results further support the notion that SVHRSP can effectively reduce senescent cells. Building upon our previous findings demonstrating the ability of SVHRSP to attenuate the expression levels of senescence markers P16 and P21 in SAMP8 mice, we utilized the H_2_O_2_-induced cell model once again to confirm whether SVHRSP is involved in regulating the cell cycle. The Western blot results demonstrate that the H_2_O_2_ group exhibited elevated protein expression levels of P16 and P21, while pre-incubation with SVHRSP reduced these elevated levels (Figure 2K–M). This suggests that SVHRSP may ameliorate age-related phenotypes. 

Based on the aforementioned results, SVHRSP has been observed to downregulate the expression of cell cycle arrest proteins P16 and P21. However, it remains unclear whether SVHRSP affects the distribution of cells in different phases of the cell cycle. To investigate this, flow cytometry was employed to determine the percentage of cells in each phase. Propidium iodide (PI), a fluorescent dye used for DNA detection, binds to double-stranded DNA and generates fluorescence intensity proportional to the DNA content. Following cellular DNA staining with PI, flow cytometry was utilized for the analysis of cell numbers in each phase of the cell cycle. The G1/G0 phase represents viable cells, the S phase indicates cells synthesizing material, and the G2/M phase signifies dividing cells. The results revealed a significantly lower number of S-phase cells in the H_2_O_2_ group compared to the control group (Figure 2N,O). Conversely, pretreatment with SVHRSP increased the number of S-phase cells when compared with the H_2_O_2_ group (Figure 2N,O). This observation suggests that H_2_O_2_ induced cells in the S phase, while SVHRSP redistributed the cell cycle and mitigated the extent of cell cycle arrest.

### 3.3. SVHRSP Can Inhibit the SIRT1/P53 Signaling Pathway, Leading to Enhanced Nuclear Translocation of NRF-2 and Subsequently Promoting the Expression of Antioxidants in SAMP8 Mice

**Figure 3:**
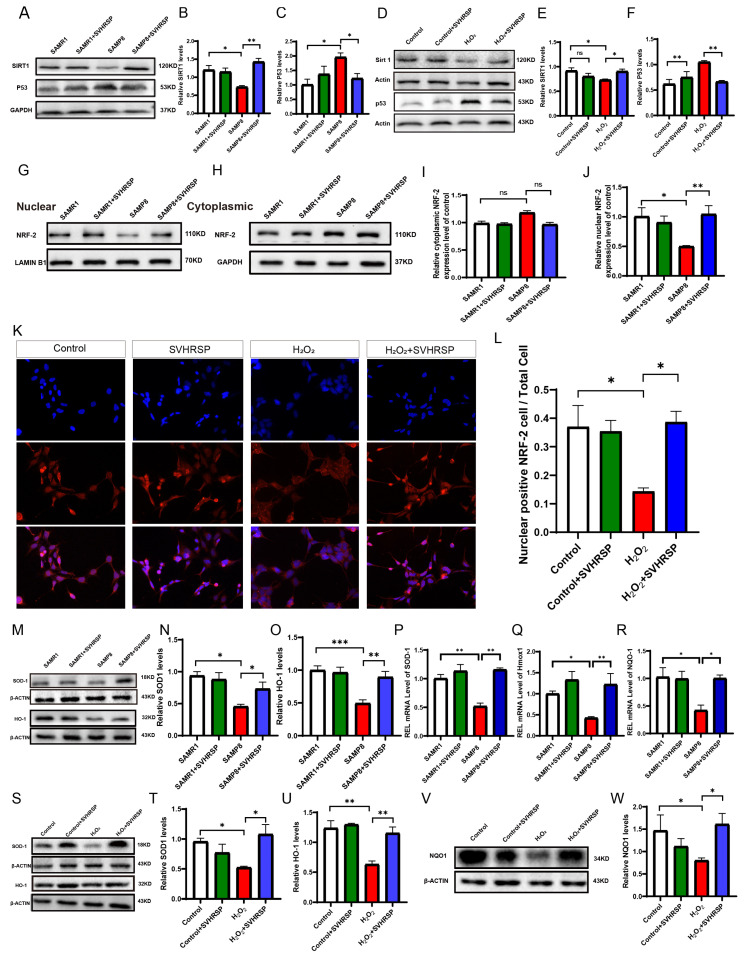
SVHRSP can inhibit the SIRT1/P53 signaling pathway, leading to enhanced nuclear translocation of NRF-2 and subsequently promoting the expression of antioxidants in SAMP8 mice. (**A**) Representative WB bands. (**B**,**C**) Quantitative analysis of SIRT1 and P53 in vivo. (**D**) Representative WB bands. (**E**,**F**) Quantitative analysis of SIRT1 and P53 in vitro. (**G**–**J**) Representative WB bands and quantification analysis of nuclear NRF2 and cytoplasmic NRF2. (**K**,**L**) Immunofluorescence images and quantification analysis of NRF2 cells/total cells (scale bar = 10 μm). (**M**) Representative WB bands. (**N**,**O**) Quantitative analysis of SOD 1 and HO-1 in vivo. (**P**–**R**) mRNA quantitative analysis of *SOD 1*, *Hmox-1,* and *NQO1* in vivo. (**S**–**W**) Representative WB bands and quantitative analysis of SOD 1, HO-1, and NQO 1 in vitro. The bars represent the mean ± SD. ns = not significant, * *p* < 0.05, ** *p* < 0.01, *** *p* < 0.001 versus the indicated groups (*n* = 3 for each group).

The SIRT1/P53 signaling pathway plays a crucial role in the regulation of various physiological processes and is closely associated with aging, DNA repair, and cell cycle control. In this study, we investigated the activation of the SIRT1/P53 signaling pathway by SVHRSP in SAMP8 mice using Western blot analysis. Our results demonstrate that the expression level of the SIRT1 protein was significantly lower in the SAMP8 model group compared to the SAMR1 control group. However, treatment with SVHRSP led to a significant upregulation in SIRT1 expression levels compared to the SAMP8 group. Furthermore, we examined the effect of SVHRSP on downstream P53 signaling pathway activation through Western blot analysis. The Western blot analysis revealed significantly higher levels of P53 in the SAMP8 model group compared to the SAMR1 control group. Interestingly, treatment with SVHRSP reduced P53 expression levels compared to the SAMP8 model group (Figure 3A–C). To further validate the impact of SVHRSP on the SIRT1/P53 signaling pathway, an SH-SY5Y cell cytotoxicity model was induced by using H_2_O_2_, and the findings were consistent with animal experiments. The Western blot analysis revealed that the expression levels of SIRT1 in the H_2_O_2_ group were significantly lower than those in the control group. In contrast, compared to the H_2_O_2_ group, pretreatment with SVHRSP resulted in a significant increase in SIRT1 expression. Subsequently, we investigated the influence of SVHRSP on downstream P53 signaling pathways regulated by SIRT1 through Western blot analysis. The Western blot analysis demonstrates a significant increase in P53 levels in the H_2_O_2_ group compared to the control group. However, pretreatment with SVHRSP significantly reduced protein expression levels of P53 compared to the H_2_O_2_ group (Figure 3D–F).

Upon exposure to oxidative stress, NRF2 translocates into the nucleus and initiates the transcription of numerous genes encoding antioxidant enzymes, thereby exerting its antioxidative function. Western blot analysis revealed that the nuclear protein expression level of NRF2 was significantly lower in the SAMP8 model group compared to the SAMR1 control group. However, treatment with SVHRSP led to a significant upregulation of nuclear protein expression of NRF2 compared to the SAMP8 model group (Figure 3G–J). To further validate the impact of SVHRSP on NRF2, an SH-SY5Y cytotoxicity model was induced with H_2_O_2_, and the nuclear translocation of NRF2 was assessed through cellular immunofluorescence. The findings reveal a decrease in the number of NRF2-positive cells within the nucleus of the H_2_O_2_ group compared to the control group. Conversely, there was an increase in the number of NRF2-positive cells within the H_2_O_2_ plus SVHRSP group compared to the H_2_O_2_ group, and this difference exhibited statistical significance (*p* < 0.05). These results indicate that SVHRSP has the potential to enhance NRF-activated nuclear translocation during senescence. (Figure 3K,L).Subsequently, we will further investigate whether this regulation promotes oxidation protein expression and subsequently reduces oxidative damage.

The downstream key molecules regulated by NRF2 include heme oxygenase-1 (HO-1), quinone oxidoreductase 1 (NQO1), and superoxide dismutase (SOD1). These enzymes play a crucial role in maintaining the balance between oxidation and antioxidation in the body, thereby protecting it from oxidative stress damage. According to the Western blot results, the expression levels of HO-1 and SOD1 were significantly decreased in the SAMP8 model group compared to the SAMR1 control group. However, treatment with NRF2 activation led to a significant increase in HO-1 and SOD1 expression levels in the treated SAMP8 group relative to the SAMP8 model group (Figure 3M–O). The mRNA expression levels of *SOD-1*, *Hmox1*, and *NQO1* were assessed. The findings reveal a significant decrease in the expression of *SOD-1*, *Hmox1*, and *NQO1* in the SAMP8 group compared to the SAMR1 group. However, following SVHRSP treatment, the expression levels of *SOD-1*, *Hmox1*, and *NQO1* returned to normal (Figure 3P–R). After conducting animal experiments, we obtained consistent results in cell experiments as well. Western blot analysis revealed significantly lower protein expression levels of HO-1, NQO1, and SOD1 in the H_2_O_2_ group compared to the control group. However, in comparison to the H_2_O_2_ model group, pretreatment with SVHRSP significantly upregulated the expression levels of HO-1, NQO1, and SOD1. Both in vivo and in vitro experiments demonstrated that SVHRSP enhanced antioxidant protein expression and played a crucial protective role against oxidative damage (Figure 3S–W).

### 3.4. SVHRSP Exerts Inhibitory Effects on Neuroinflammation in SAMP8 Mice by Modulating the MAPKs/NF-κB Signaling Pathway

**Figure 4:**
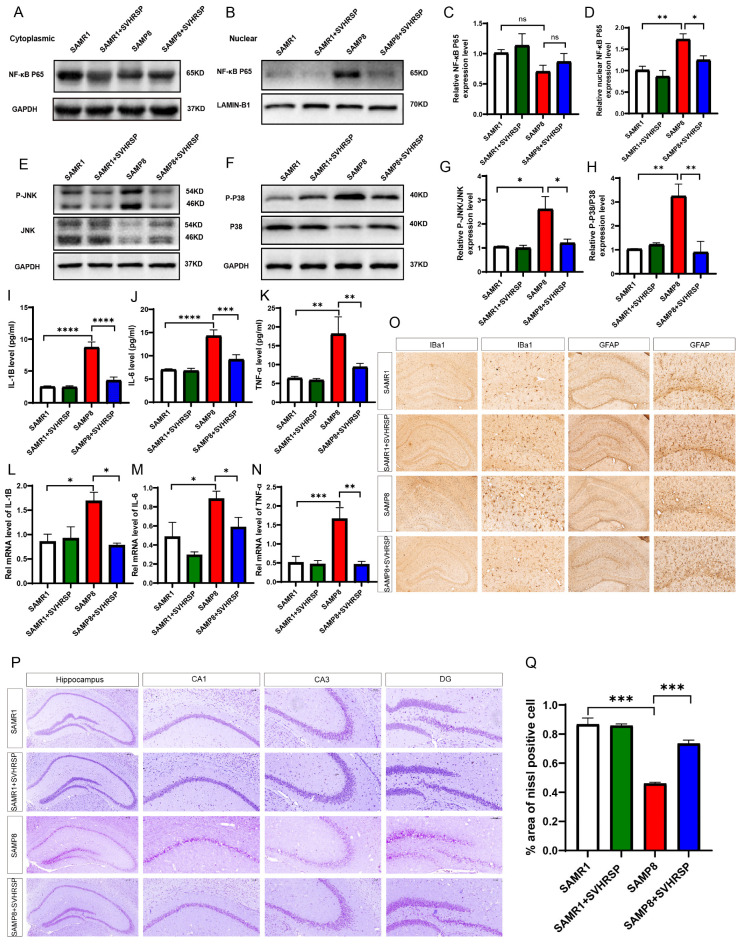
SVHRSP exerts inhibitory effects on neuroinflammation in SAMP8 mice by modulating the MAPKs/NF-κB signaling pathway. (**A**–**D**) Representative Western blot bands and quantitative analysis of cytoplasmic NF-κB p65 and nuclear NF-κB p65. (**E**–**H**) Representative Western blot bands and quantification analysis of p-JNK, JNK, p-P38, and P38. (**I**–**K**) Quantitative analysis of the serum level of *IL-1B*, *IL-6,* and *TNF-α* measured by using ELISA. (**L**–**N**) Quantitative analysis of the mRNA level of *IL-1B*, *IL-6,* and *TNF-α* measured by using QRT-PCR. (**O**) Representative immunohistochemical images of Iba-1 (left) and GFAP (right). (**P**,**Q**) Representative image and quantitative analysis of Nissl staining. The bars represent the mean ± SD. ns = not significant, * *p* < 0.05, ** *p* < 0.01, *** *p* < 0.001, **** *p* < 0.0001 versus the indicated groups. Scale bar = 50 μm (*n* = 3 for each group).

The reported findings suggest that SIRT1 exerts inhibitory effects on NF-κB transcription and attenuates the expression of downstream factors. Notably, the intracellular MAPKs/NF-κB signaling pathway is implicated in regulating the transcription and expression of proinflammatory factors. We assessed the activation of NF-κB signaling in SAMP8 mice treated with SVHRSP by using Western blot analysis. The results revealed a reduction in the cytoplasmic matrix expression of the NF-κB P65 protein in the hippocampus of SAMP8 mice compared to the control group. However, treatment with SVHRSP exhibited an increase in expression, albeit without statistical significance. Conversely, an upregulation in nuclear expression of the NF-κB P65 protein was observed in the hippocampus of SAMP8 mice, which was effectively mitigated by SVHRSP. Subsequently, we examined two key molecules c-Jun N-terminal kinase (JNK) and P38 lightning phosphorylation levels within the MAPK pathway in the hippocampus. The findings demonstrate increased levels of JNK and P38 phosphorylation levels in SAMP8 mice; however, treatment with SVHRSP led to the inhibition of both JNK and P38 phosphorylation, as illustrated in Figure 4A–H. These results indicate that SVHRSP exerts inhibitory effects on the MAPKs pathway. 

ELISA and Q-PCR were employed to assess the levels of *IL-1B*, *IL-6*, and *TNF-α* in both serum and brain tissue. Statistical analysis revealed a significant increase in the levels of *IL-1B*, *IL-6*, and *TNF-α* in the SAMP8 group compared to the control group (Figure 4I–N). Furthermore, SVHRSP intervention demonstrated a noteworthy reduction in these cytokine levels within both serum and brain tissue when compared to the model group. These findings indicate that SVHRSP intervention effectively mitigates peripheral blood and brain inflammatory responses.

Subsequently, astrocytes and microglia were immunohistochemically labeled using GFAP and Iba-1 antibodies, respectively. The results demonstrate that in the hippocampus of control SAMR1 mice, microglia exhibited intact cell morphology characterized by small cell bodies and processes. However, in the model group of SAMP8 mice, there was an observed increase in the volume of microglia in the hippocampus along with irregular and flat polygonal shapes, elevated numbers, and enhanced staining intensity. In contrast, treatment with SVHRSP significantly reduced both the number and aggregation of microglia, indicating its inhibitory effect on microglial activation. Furthermore, astrocytes in the hippocampus of SAMR1 mice from the control group displayed intact morphology characterized by small cell bodies and slender processes. Conversely, in the model group of SAMP8 mice, there was an enlargement in astrocyte volume accompanied by abnormally thickened processes as well as increased numbers and intensified staining. Notably, however, the administration of SVHRSP resulted in a reduction in astrocyte count while also inducing smaller cell bodies and slenderer processes—suggesting its ability to inhibit astrocytic activation (Figure 4O).

The proper functioning of numerous vital physiological processes in the nervous system relies on the intricate communication between interconnected neurons. Nissl staining, a fundamental dye that selectively highlights basophilic substances known as Nissl bodies within neurons, enables the detection of neuronal morphology and quantification in mouse brains, thereby enabling the observation of neuronal loss. Our findings reveal a significant reduction in the number of Nissl body-positive cells in SAMP8 mice compared to SAMR1 mice. However, treatment with SVHRSP significantly augmented the population of Nissl body-positive cells in SAMP8 mice compared to the SAMP8 model group, thus indicating its potential for reducing neuronal damage (Figure 4P,Q).

## 4. Discussion

The process of aging is an inherent physiological phenomenon which progressively impacts human cognitive function, metabolic processes, and immune system. Given the imminent advent of an aging society, extensive research on anti-aging methods has assumed paramount significance. Bioactive compounds derived from plants and animals hold great promise as potential sources for the development of pharmaceuticals targeting anti-aging conditions. Previous studies have demonstrated that SVHRSP has protective effects on dopamine neurons in 6-OHDA-induced PD mouse model mice by reducing neuroinflammation [26,27,28] and upregulating the expression of brain-derived neurotrophic factor (BDNF) [16]. In addition, it significantly enhances resistance to oxidative stress and heat stress by activating the IGF-1-repressed anti-aging nuclear factor Daf16/FOXO [15]. Cognitive learning and memory dysfunction are important behavioral changes in the aging process. Previous laboratory findings have demonstrated that SVHRSP can mitigate cognitive dysfunction and enhance synaptic plasticity in SAMP mice. However, the underlying mechanisms by which SVHRSP improves cognitive function in these mice remain unexplored. In this study, we have identified SVHRSP as a potential therapeutic agent for reversing aging-related phenotypes both in vivo and in vitro, particularly by conferring protection against oxidative damage during aging through the activation of the NRF2 transcription factor. Moreover, our results indicate that SVHRSP reduces cell cycle arrest and senescent cells by regulating cell cycle distribution. We postulate that SVHRSP may exert a protective role against aging by activating the SIRT1-P53 signaling pathway to promote nuclear translocation of NRF2 for reducing stress levels, as well as inhibiting the MAPKs/NF-κB signaling pathway to decrease inflammatory factor release.

The SAMP8 mice serve as a valuable mouse model for accelerated aging and a shortened lifespan, enabling the study of age-related cognitive decline. Previous research has indicated that these mice exhibit impaired learning and memory function as early as 4 months of age [29]. Following a 12-week treatment with SVHRSP in 4-month-old SAMP8 mice, an assemblage of behavioral tests was conducted to assess their cognitive abilities. Notably, the administration of SVHRSP significantly improved short-term spatial memory performance in the Y-maze test. Moreover, the passive avoidance test, widely employed to evaluate memory and cognition-enhancing effects, demonstrated that SVHRSP effectively enhanced memory and cognitive performance compared to control conditions. In the novel object recognition test, SVHRSP administration resulted in a significant increase in both the discrimination index (DI) of SAMP8 mice and their exploration time in finding the novel object. The Morris water maze task, a classic assessment of spatial memory, evaluates mice in terms of their ability to escape from water based on environmental cues. On the fifth day of navigation trials, mice treated with SVHRSP exhibited reduced escape latencies and swimming distances along with increased preference for searching within the target quadrant. During probe trials, SVHRSP significantly decreased latency to reach the platform while promoting a higher frequency of entries crossing it; additionally, it increased both the duration and distance of searching within the target quadrant. Overall, these findings demonstrate that SVHRSP ameliorates cognitive performance deficits observed in SAMP8 mice across various behavioral paradigms.

Synaptic plasticity, a fundamental biological mechanism underlying learning, memory, and cognitive function [30,31], refers to the ability of synapses to efficiently transmit nerve activity changes. It is well established that synaptic plasticity deficiency is implicated in cognitive impairment associated with neurodegenerative diseases [32]. The reduction in synaptic plasticity observed in the aging hippocampus is generally believed to be associated with cognitive decline [33]. Therefore, it is crucial to explore promising targets for enhancing synaptic plasticity in neurodegenerative disorders. Long-term potentiation (LTP), which represents the most dominant form of synaptic plasticity, has been strongly correlated with memory formation and retention. Its impairment closely correlates with cognitive deficits. In our study, we measured the slope and amplitude of fEPSP as reference values for LTP (the fEPSP slope reflects the rate of excitatory synaptic response to stimuli, while the amplitude indicates the intensity of synaptic stimulation). Our results indicate that the administration of SVHRSP can enhance synaptic transmission by improving LTP through an increase in both the amplitude and slope of fEPSP, as compared to the SAMP8 group. Morphological analysis revealed that SVHRSP treatment restored damaged presynaptic and postsynaptic components as well as increased the content of synaptic vesicles. These findings demonstrate that SVHRSP could inhibit structural damage at synapses in the hippocampus of SAMP8 mice. SYN is widely used for studying synaptogenesis and synaptic function [34]. After phosphorylation, SYN, the most abundant presynaptic vesicle transmembrane protein, binds to synaptic vesicles to regulate their movement and release in nerve endings, thereby modulating neurotransmitter release [35]. PSD95 is widely expressed in excitatory glutamatergic synapses’ postsynaptic membranes and its reduction indicates damage to postsynaptic density. In this study, SVHRSP significantly increased SYN and PSD95 expression in SAMP8 mice, suggesting a protective effect and improvements in memory in aging mice.

The accumulation of senescent cells progressively increases with age in the body, and this accumulation to a certain extent leads to the degeneration of nervous system function. According to research reports, the proportion of senescent cells in the hippocampus of 6-month-old mice was 14.57 ± 2.74%, which doubled to 31.66 ± 14.12% at 18 months old, and reached 50.76 ± 14.41% in 24-month-old mice. The presence of a substantial population of senescent-positive cells in aged mice may have an impact on nervous system function to some extent. Several studies have demonstrated that transplantation of a small number of senescent cells into young mice can induce physical dysfunction, while elimination of these cells can alleviate physical dysfunction in aged mice and increase their remaining lifespan. Timely elimination of senescent cells may be considered an important strategy for anti-aging [36]. The SA-β-gal staining assay has been widely employed as a biological marker for aging cells [36]. We utilized flow cytometry to assess the percentage of cell volume at different time points. Propidium iodide (PI) is a fluorescent dye used for DNA detection, and its fluorescence intensity is proportional to the DNA content. After intracellular DNA staining with PI, we analyzed cell numbers in each cell cycle phase based on their DNA content by using flow cytometry. The G1/G0 phase represents living cells, the S phase indicates material synthesis, and the G2/M phase reflects the number of cell divisions. In this study, SVHRSP not only reduced brain SA-β-gal activity but also alleviated G0/G1-phase cell cycle arrest in SAMP8 mice. Cellular senescence can manifest as cell cycle arrest without cell division, and P16 and P21 can be regarded as protein markers for cell cycle arrest, which are crucial indicators of cellular senescence. These findings suggest that SVHRSP may alleviate the state of cellular senescence in SH-SY5Y neuronal cells induced by H_2_O_2_ and in SAMP8 mice, indicating that SVHRSP could reduce the expression of aging markers.

Sirtuins are a family of NAD+-dependent deacetylases that have been highly conserved throughout evolution [37]. Substantial evidence suggests that SIRT1 is involved in numerous biological processes, including the regulation of cell cycle, DNA repair, inflammation [38], aging [39], and protection against neurodegenerative diseases [40]. Previous studies have demonstrated that increased expression of the SIRT1 gene extends the lifespan in Caenorhabditis elegans [8]. Similarly, overexpression of the SIRT1 homolog in mice can promote lifespan extension [9]. Therefore, SIRT1 has garnered significant interest as a regulator of longevity. As a non-histone substrate of SIRT1, P53 activation has been implicated in apoptosis, cell cycle arrest, and aging [41,42]. P21 is known to be a target of P53 and its expression regulates cell cycle arrest during cellular senescence. Acetylation of P53 by SIRT1 reduces the expression of P21 [38] and inhibits DNA damage- and stress-mediated cellular senescence [43]. Pretreatment with SVHRSP activates SIRT1 and inhibits P53 to attenuate cell injury; thus, it may serve as a potential therapeutic strategy for aging-related diseases.

The regulation of oxidative stress is influenced by multiple factors, among which NRF2 plays a crucial role as a transcription factor in this process. Under normal physiological conditions, NRF2 activity is suppressed and remains localized in the cytoplasm. However, upon the occurrence of oxidative stress, NRF2 can initiate a cascade of antioxidant and detoxification genes to safeguard the organism against the deleterious effects of reactive oxygen species (ROS) [44,45]. The cyclin-dependent kinase (CDK) inhibitor P21 plays a crucial physiological role in the regulation of NRF2 expression. Multiple studies have demonstrated that P21 can interact with NRF2 and disrupt its binding to Keap1, thereby impeding the degradation of NRF2 [46,47]. Additionally, NRF2-mediated oxidative stress also contributes to cell cycle regulation by inactivating the P16/pRb signaling pathway [48]. In addition to that, NRF2 has close associations with aging. Studies have reported that the downregulation of NRF2 in the hippocampus of SAMP8 mice triggers oxidative stress and neuroinflammation, thereby resulting in cognitive dysfunction [44,45]. Activation of the NRF2 signaling pathway has been shown to ameliorate AD-related pathological characteristics in SAMP8 mice [44,45]. Therefore, we investigated whether SVHRSP could induce the nuclear translocation of the NRF2 transcription factor in the brains of SAMP8 mice by using karyoplasmic separation techniques. Our results demonstrate an increase in nuclear protein levels of NRF2 in the SAMP8 plus SVHRSP group, while cytoplasmic protein levels remained unchanged. Consistent findings were observed through cellular immunofluorescence analysis for H₂O₂-induced cells regarding the nuclear translocation of NRF2. In conclusion, SVHRSP can mitigate age-related oxidative stress by promoting the nuclear translocation of NRF2. To further validate the role played by NRF2 in SVHRSP’s anti-aging effects, we assessed the expression levels of downstream antioxidant proteins regulated by this transcription factor including SOD1, NQO1, and HO-1. The Western blot results clearly demonstrate that SVHRSP effectively enhanced the expression of SOD1 and HO-1 proteins in the brain of SAMP8 mice. Concurrently, the findings from the in vitro cell experiments were consistent with those obtained in vivo, as SVHRSP significantly upregulated the expression of SOD1, NQO1, and HO-1 proteins in an H_2_O_2_-induced cellular model. These results provide evidence that SVHRSP facilitates NRF2 activation, promotes downstream antioxidant protein expression, and mitigates oxidative damage during aging. The present findings suggest that the protective effect of SVHRSP in aging may be achieved through activation of the SIRT1-P53 signaling pathway. The deacetylation of PPARγ coactivator-1α (PGC-1α) can be facilitated by SIRT1, thereby promoting the subsequent upregulation of NRF2 and antioxidation-related genes [31,32]. Furthermore, we hypothesized that SVHRSP activates the SIRT1-P53 signaling pathway and regulates the activation of the NRF2 transcription factor to provide protection against oxidative stress and delay the aging process.

Both oxidative stress and inflammatory responses are crucial components in the pathogenesis of neurodegenerative diseases. Accumulating evidence suggests that oxidative stress is closely associated with the inflammatory response. The NF-κB signaling pathway in the central nervous system plays a vital role in regulating various functions, including neuronal plasticity and growth [48]. Moreover, SIRT1 signaling negatively regulates NF-κB activity [49]. Therefore, our study focused on exploring the potential of inhibiting NF-κB as a novel therapeutic target for age-related diseases [50]. Yeung et al. demonstrated that SIRT1 interacts with the RelA/p65 subunit of NF-κB. Since acetylation of p65 enhances the transcriptional activity of the NF-κB complex, SIRT1-mediated deacetylation suppresses NF-κB signaling [51]. SVHRSP treatment effectively enhances SIRT1 activity and significantly suppresses the phosphorylation of NF-κB p65, leading to a remarkable reduction in the expression of immune genes encoding cytokines such as *IL-1B*, *TNF-α*, and *IL-6*. Aging is known to induce the phosphorylation of NF-κB and members of MAPK family, such as JNK and P38-MAPK [52]. Therefore, targeted inhibition of NF-κB/MAPK phosphorylation plays a crucial role in preventing age-induced neural degradation. In this study, Western blot analysis revealed that aging models exhibited significant increases in the phosphorylation of P38, JNK, and p65, along with decreased protein levels of SIRT1; however, these changes were effectively inhibited by SVHRSP treatment. Henceforth, we infer that the SIRT1-NF-κB signaling pathway contributes to the anti-senescence effects of SVHRSP on neurodegenerative diseases associated with aging.

The hypothesis that glial cell initiation induces a brain inflammatory response is a well-established mechanism of aging. In this study, the immunohistochemical experiment demonstrated a significant increase in astrocytic and microglial activation in SAMP8 mice, which aligns with previous research [53,54]. In aging animal models, glial activation leads to degenerative neuronal lesions. All of these findings suggest that regulating neuroinflammation could be a potential therapeutic target. SVHRSP intervention effectively reduced astroglial and microglial activation in SAMP8 mice. Microglia can undergo polarization into either the M1 proinflammatory phenotype or the M2 anti-inflammatory phenotype in response to various microenvironmental perturbations. The former elicits inflammation and neurotoxicity, while the latter promotes anti-inflammation and neuroprotection. Both phenotypes play crucial roles in the pathogenesis of neurodegenerative diseases, thus rendering microglia a double-edged sword in such conditions. Precise regulation of microglial activation is indispensable for maintaining normal microglial function, ensuring brain homeostasis, and preventing neurodegenerative diseases [55]. Transient priming of microglia is generally considered neuroprotective; however, prolonged exposure to inflammatory stimuli can result in the release of various cytotoxic molecules such as reactive oxygen species, chemokines, and proinflammatory factors. The levels of proinflammatory factors released by M1 microglia, such as *TNF-α*, *IL-1B*, and *IL-6*, were significantly elevated in SAMP8 mice. However, the intervention with SVHRSP resulted in a decrease in the expression of these proinflammatory factors. Astrocytes play a crucial role in either inhibiting or promoting neurodegeneration through their interaction with extracellularly released molecules and the shared microenvironment with neurons. Inflammatory factors released by microglia, including *TNF-α*, *IL-1B*, and *IL-6*, can directly activate astrocytes and induce the production of oxygen free radicals that contribute to neuronal damage [56]. Additionally, another study demonstrated that improved neuronal deactivation was accompanied by a reduction in astrocyte priming.

Neurons serve as the fundamental functional units within the intricate neural network of the brain, and excessive neuronal loss can detrimentally impact cognitive and other essential functions [57]. The Nissl body, a distinctive cellular structure reflecting neuronal function and aiding in protein synthesis, was found to exhibit damage in hippocampal and cortical neurons of SAMP8 mice. Additionally, disorganized arrangement of vertebral body cells and a reduced number of normal cells were observed. Notably, these alterations were effectively reversed by SVHRSP treatment, suggesting a potential neuroprotective effect associated with its anti-aging properties in SAMP8 mice.

**Figure 5:**
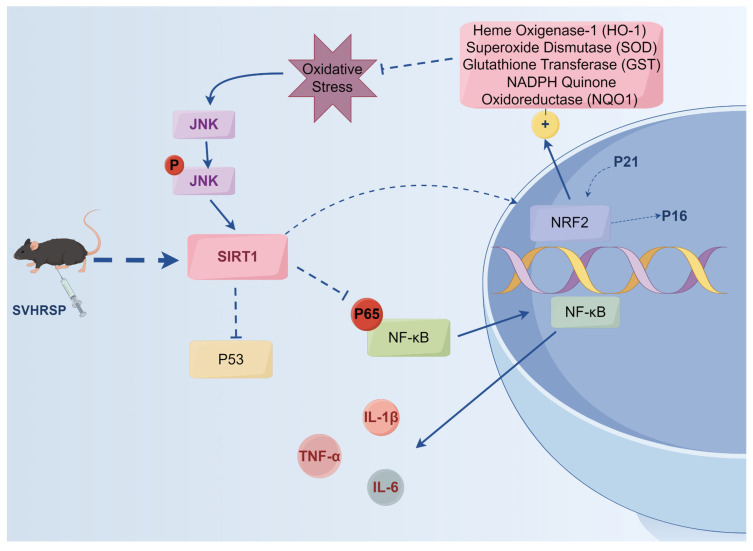
SVHRSP alleviates age-related cognitive deficiency by reducing oxidative stress and neuroinflammation. SVHRSP can enhance the expression of the SIRT1 protein, reduce P53 activity, promote the nuclear translocation of the NRF2 transcription factor, and induce the expression of antioxidant enzymes such as SOD1, NQO1, and HO-1 to counteract oxidative stress. Furthermore, SVHRSP inhibits the expression of senescence markers P16 and P21 proteins. Additionally, by suppressing NF-κB pathway activation, SVHRSP suppresses the release of inflammatory factors IL-1β, TNF-α, and IL-6. (The graphical abstract is depicted by Figdraw 2.0.)

The effects of SVHRSP on age-related cognitive deficits, including the reduction in oxidative stress and neuroinflammatory responses through the Sirt 1 pathway, are summarized in Figure 5.

## 5. Conclusions

Our study suggests that SVHRSP plays a crucial role in the SIRT1-P53 signaling pathway during aging. SVHRSP enhances the expression of the SIRT1 protein, reduces P53 activity, and facilitates the translocation of the NRF2 transcription factor into the nucleus. This leads to an antioxidant effect by promoting the expression of antioxidant enzymes such as SOD1, NQO1, and HO-1 proteins. Additionally, SVHRSP inhibits the release of inflammatory agents by suppressing the NF-κB pathway and attenuates astrocytic and microglial activation. Moreover, SVHRSP suppresses the expression of senescence markers P16 and P21 proteins, thereby improving senescence-related phenotypes and reducing the neuronal loss associated with cognitive impairment. These findings suggest that SVHRSP may hold promise as a therapeutic intervention for age-related cognitive decline.

## Data Availability

The data are available upon request from the corresponding author.

## Supplementary Materials

The following supporting information can be downloaded at: https://www.mdpi.com/article/10.3390/antiox13060628/s1, Figure S1: SVHRSP reduces H_2_O_2_-induced oxygen radical production in SY5Y cells.
