# Supplementary material for: SVHRSP Alleviates Age-Related Cognitive Deficiency by Reducing Oxidative Stress and Neuroinflammation
Source: Antioxidants (Basel). 2024 May 21;13(6):628. doi: 10.3390/antiox13060628 (PMC11200511; doi:10.3390/antiox13060628)
Supplement: Supplementary file 1 [file antioxidants-13-00628-s001.zip › antioxidants-2968217-supplementary.pdf]

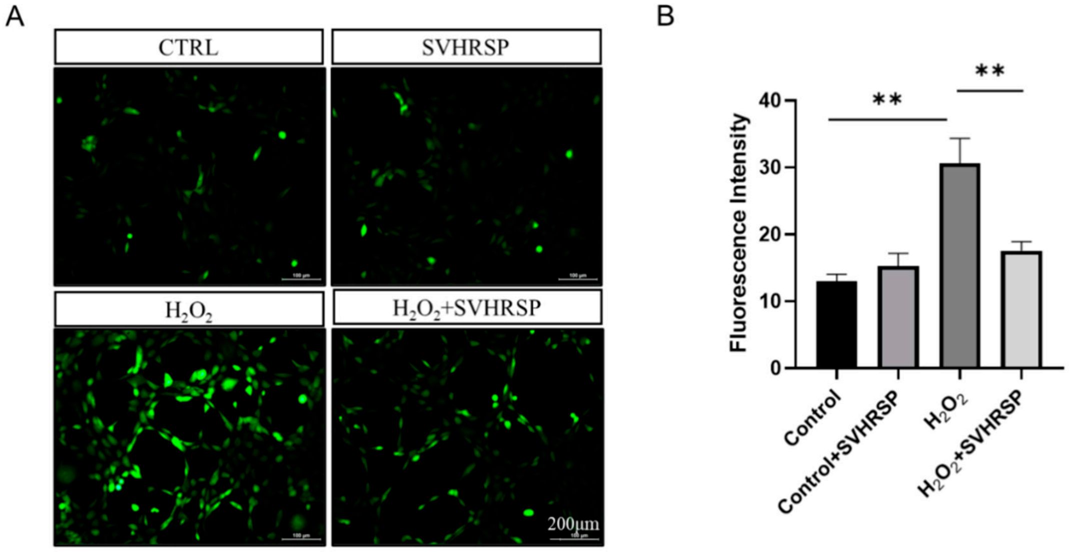

Figure S1. SVHRSP reduces H_2O_2-induced oxygen radical production in SY5Y cells. (A) Representative immunofluorescent images of ROS in H_2O_2-induced cell model. ROS were labeled with DCFH-DA (green). (B) qualitative analysis of the number of ROS positive cells. Data were presented as means ± S. E. M. n=3. (**P*<0.05, ***P*<0.01)
